# Craft Non-Alcoholic and Low-Alcohol Beer (NABLAB): Perceived Role as Functional Foods Among Italian Consumers and a Focus on Benefits for Well-Being and Physical Activity

**DOI:** 10.3390/nu18010033

**Published:** 2025-12-21

**Authors:** Mario Ruggiero, Nicla Mercurio, Leopoldo Ferrante, Olga Scudiero, Filomena Mazzeo

**Affiliations:** 1 Department of Medical, Human Movement and Well-Being Sciences, University of Naples Parthenope, Via Medina 40, 80133 Napoli, Italy; 2 Department of Humanities and Social Sciences, University of Sassari, Via Roma 151, 07100 Sassari, Italy; nmercurio@uniss.it; 3 Department of Economics, Law, Cybersecurity and Sports Sciences, University of Naples Parthenope, 80035 Nola, Italy; leopoldo.ferrante@collaboratore.uniparthenope.it; 4 Department of Molecular Medicine and Medical Biotechnologies, Federico II University, Via Sergio Pansini 5, 80131 Napoli, Italy; olga.scudiero@unina.it; 5 CEINGE-Biotecnologie Avanzate Franco Salvatore, Via G. Salvatore 486, 80145 Napoli, Italy; 6 Task Force on Microbiome Studies, University of Naples Federico II, 80100 Napoli, Italy

**Keywords:** craft beer, non-alcoholic and low-alcohol beer (NABLAB), functional foods, nutraceuticals, nutrition, sports supplements, wellness, consumers’ perception, fermented and healthy beverages, athletes

## Abstract

Background/Objectives: Craft non-alcoholic and low-alcohol beer (NABLAB) is attracting increasing attention as potential functional beverages due to their content of bioactive compounds such as polyphenols, vitamins, and minerals, and their suitability for health-oriented lifestyles. This study investigated Italian consumers’ perceptions of craft NABLAB and explored possible generational differences in their acceptance. Methods: A descriptive cross-sectional online survey was conducted between March 2024 and March 2025 among adults living in Italy. The questionnaire, composed entirely of closed-ended questions, investigated familiarity with craft NABLAB, attitudes toward their potential health-related properties, and willingness to recommend them. Results: A total of 527 valid responses were analyzed descriptively and grouped by generation (Generation Z, Millennials, Generation X, and Baby Boomers). Results showed that 68.3% of participants would recommend craft NABLAB to others interested in their functional properties, while 55.0% reported higher motivation to purchase when informed about their potential health benefits. Familiarity with these products remained limited (34.7% had tried them, and only 22.2% considered them easy to find). Baby Boomers and Millennials were more receptive, possibly due to greater health awareness and openness to innovation, whereas Generation Z displayed curiosity despite lower consumption experience. Conclusions: Overall, Italian consumers show a growing interest in craft NABLAB, especially when linked to wellness and active lifestyle benefits. Enhancing product availability and communication focused on health and functionality could promote more moderate and conscious drinking habits, contributing to a gradual cultural shift toward reduced alcohol consumption.

## 1. Introduction

Beer is one of the most ancient and widely consumed fermented beverages, with origins dating back to the Neolithic era and documented production in Mesopotamia and Egypt [1,2,3]. Over the centuries, beer has continuously adapted to market dynamics and cultural trends. In recent decades, the so-called *Beer Renaissance* has fostered the expansion of craft brewing worldwide, particularly in Europe and North America, as consumers increasingly seek authenticity, diversity of styles, and higher product quality [4,5,6]. At the same time, sustainability has become a defining value of the modern craft beer movement. The recovery and reuse of brewing by-products, such as brewer’s spent grain (BSG), reflect a circular-economy approach that reduces environmental impact while generating high-value ingredients rich in proteins, fibers, and phenolic compounds with potential nutraceutical applications [7,8,9,10].

The differences with industrial beer are that the craft beer must be produced by small independent breweries and cannot undergo pasteurization or microfiltration [11,12,13]. These features preserve its sensory complexity and bioactive compounds, which are often reduced in industrial processing. As a result, craft beer retains a richer nutraceutical profile, including B-group vitamins, proteins, essential minerals such as potassium, magnesium, and selenium, and a wide variety of polyphenols with antioxidant and anti-inflammatory activity [14,15,16]. Evidence has suggested beneficial effects on cardiovascular health, neuroprotection, and modulation of the gut microbiota, supporting the growing academic and commercial interest in craft beer as a beverage with added nutritional value [17,18]. Moreover, recent evidence has highlighted the relevance of the main bioactive compounds found in craft beer, particularly polyphenols such as xanthohumol, isoxanthohumol, and prenylnaringenins, which exert antioxidant and anti-inflammatory effects capable of reducing oxidative stress [19,20] and supporting endothelial function [21,22]. These compounds have also been associated with potential cardioprotective [23,24], neuroprotective [25,26], and metabolic benefits, including the modulation of glucose and lipid homeostasis [27,28], and possible positive effects on bone metabolism [29,30]. Nevertheless, the presence of ethanol limits the extent to which beer can be considered a functional beverage. Alcohol consumption is contraindicated in several population groups, including pregnant women, drivers, and individuals with specific health conditions, and excessive intake is a major risk factor for liver disease, social dysfunction, and reduced productivity [31]. To overcome these barriers, non-alcoholic and low-alcohol beers (NABLAB), both industrial and craft, have emerged as a rapidly growing segment. Importantly, the absence of alcohol not only eliminates the risks associated with ethanol but may also improve the nutritional profile of beer, preserving its [19,20] vitamins, minerals, and polyphenolic compounds while enhancing its suitability for wider populations [32,33]. These features position craft NABLAB within the broader framework of functional foods and beverages, especially in contexts related to health, wellness, and sports recovery [34]. Functional foods are defined as products that, beyond their basic nutritional value, contain bioactive components capable of providing additional health benefits or contributing to the prevention of certain diseases. They play a growing role in promoting overall well-being and in supporting physiological functions, including immune, cardiovascular, and metabolic health [35,36,37,38]. In this perspective, the promotion of healthy and balanced dietary patterns, integrating functional foods and beverages, represents a key strategy for disease prevention and the maintenance of long-term health [39,40,41,42,43].

In addition, growing attention has been devoted to the potential role of beer, and especially craft NABLAB, in sports and wellness contexts. Craft NABLAB is rapidly gaining popularity among athletes and fitness enthusiasts, moving from a niche product to a recognized post-exercise recovery beverage. The presence of electrolytes such as potassium and magnesium, together with carbohydrates and polyphenols, supports hydration and recovery after physical activity [14,15]. The appeal lies in the ability to enjoy the ritual and flavor of beer without the negative, dehydrating, and performance-impairing effects of alcohol. Craft NABLAB has been shown to reduce exercise-induced inflammation and oxidative stress, while contributing to immune modulation [34]. The main advantages of craft NABLAB for athletes, summarized in Table 1.

These properties make craft NABLAB an attractive alternative to conventional sports drinks, combining consumer appeal with possible functional benefits. Moreover, attention to what athletes can safely consume remains essential in the context of sports nutrition and anti-doping policies [48,49]. Alcohol (ethanol) was previously listed by the World Anti-Doping Agency (WADA) Prohibited List for specific disciplines, with a violation threshold of 0.10 g/L, but was removed in 2018 [50]. Nonetheless, its consumption remains discouraged in both professional and amateur sport due to its negative effects on coordination, recovery, and hydration [51,52,53]. Education on anti-doping principles is essential to promote ethical behavior and health protection among athletes [54,55].

In this context, NABLAB offers an alcohol-free option aligned with health promotion and responsible consumption in athletic settings. Previous studies show that functional foods and beverages can support recovery, reduce inflammation, and improve performance [56,57,58,59]. The combination of regular physical activity and the consumption of functional foods represents a key strategy for maintaining health and preventing chronic diseases. Together, they contribute to improving metabolic efficiency, supporting recovery processes, and promoting overall physical and psychological well-being [60,61,62,63,64].

In recent years, Italy’s craft beer sector has seen rapid growth, successfully carving out a place for itself despite the country’s strong wine-oriented tradition [65,66,67]. Consumer acceptance, however, remains a critical issue. Previous evidence suggests that younger cohorts are more willing to experiment with innovative beer styles, associating them with quality and lifestyle values [68]. Understanding how different generations perceive craft NABLAB is therefore essential to assess its realistic potential as a functional beverage in the Italian context.

Despite the growing interest in craft NABLAB, little is known about how consumers—particularly across different generations—perceive their nutraceutical value, especially in countries traditionally oriented toward wine consumption such as Italy. Existing studies rarely integrate product attributes with consumer-behavior frameworks, leaving unclear how health beliefs and lifestyle orientations influence the acceptance of craft NABLAB. This gap highlights the need for empirical research linking consumer perceptions, functional positioning, and generational differences.

The present study examines Italian consumers’ perceptions of craft NABLAB, with a focus on generational segmentation (Generation Z, Millennials, Generation X, and Baby Boomers). Specifically, it aims to assess whether these beverages are regarded merely as craft NABLAB alternatives to conventional beer or as products with genuine nutraceutical properties, including potential benefits in wellness and sports contexts, that could support their inclusion within the framework of functional nutrition.

## 2. Materials and Methods

This study employed a descriptive cross-sectional design to investigate Italian consumers’ perceptions of craft NABLAB, with a particular focus on their potential positioning as functional beverages and their role in wellness and sports contexts. A structured online survey was developed to capture multidimensional data, including demographic characteristics, consumption patterns, attitudes toward functional properties, and generational differences in consumer behavior.

### 2.1. Study Design and Participants

Participants were eligible for inclusion if they were of legal drinking age in Italy (≥18 years old), resided in Italy, and provided informed consent to complete the online questionnaire. Only fully completed questionnaires were included in the analysis.

Exclusion criteria included incomplete responses, participants under 18 years of age, and questionnaires submitted more than once by the same individual (duplicates identified and removed before analysis).

Generational segmentation was a key element of the study design, with respondents categorized into four cohorts according to widely accepted definitions: Generation Z (born 1997–2012), Millennials (1981–1996), Generation X (1965–1980), and Baby Boomers (1946–1964) [69]. This stratification allowed for comparison of attitudes across age groups, given prior evidence that younger consumers are more willing to experiment with innovative beer styles, while older generations tend to show more traditional consumption patterns. In total, 527 surveys were collected.

### 2.2. Data Collection Procedure

A structured, self-administered online questionnaire was created and shared via Google Forms. The survey link was disseminated through various channels, including social media platforms (e.g., Facebook, Instagram), online craft beer communities, and university mailing lists, to maximize participant diversity across different age groups and regions of Italy. A convenience sampling method was used, allowing quick recruitment and covering different demographic segments.

Data collection was conducted over 12 months, from March 2024 to March 2025. Before the survey was initiated, participants were provided with an electronic information sheet outlining the purpose of the study, the voluntary nature of their participation, and assurances of data confidentiality. Informed consent was obtained electronically before accessing the questionnaire.

The study employed a convenience, self-selected sample, as participants voluntarily chose to complete the online questionnaire. This sampling approach may introduce self-selection bias, which should be considered when interpreting the results.

The research protocol was reviewed and approved by the Ethics Committee of the School of Medicine at the University of Naples Federico II (protocol no. 200/17), in accordance with the Declaration of Helsinki. All data were collected anonymously, and no personal identifiers were kept.

### 2.3. Survey Instrument and Content

The structured, self-administered questionnaire was designed to explore Italian consumers’ perceptions of craft NABLAB and their potential positioning within the category of functional products. The questions were closed-ended, formulated in dichotomous (yes/no) or multiple-choice format.

The first part of the questionnaire collected socio-demographic information, which is useful for describing the sample and grouping participants by generation. The variables included age, sex, and educational level.

The second section covered the main questionnaire and aimed to examine several aspects: beer consumption habits and familiarity with craft NABLAB; perceptions and attitudes toward the health-related and functional properties of beer; and willingness to buy and spend, including the willingness to pay a higher price for products with nutraceutical features.

Before data collection, the questionnaire was tested on a pilot group (*n* = 20) to ensure clarity, internal consistency, and ease of completion. This step was conducted to verify that the questions were interpreted as intended and to identify any ambiguous wording. No major structural changes were required based on this feedback. It is important to note that this pre-test was a feasibility check and not a formal psychometric validation study. Participation was anonymous, voluntary, and not incentivized.

### 2.4. Data and Statistical Analysis

The statistical analysis was conducted in two stages. First, descriptive statistics were calculated for the entire sample and for the generational subgroups using Microsoft Excel. This included computing the absolute number (*n*) of participants for each generation (Generation Z, Millennials, Generation X, and Baby Boomers) and for the overall sample, as well as the corresponding percentages (%) for each response to every question. These descriptive data were used to summarize the sample characteristics and to construct the contingency tables for the subsequent analyses.

In the second stage, to examine the association between generational group (independent variable) and questionnaire responses (dependent variables), the MATLAB R2025b software was employed. For each multiple-choice question, a Chi-square (χ^2^) Test of Independence was applied to the *r* × *c* contingency tables to assess the association between generational cohort (independent variable) and each categorical survey response (dependent variable). This test assessed the statistical significance of the association by calculating the χ^2^ statistic, degrees of freedom (*df*), and the corresponding *p*-value. To evaluate the strength of the association (effect size), Cramér’s V Coefficient was also computed for all questions. Statistical significance was set at α = 0.05.

## 3. Results

The main findings of the survey are organized into two parts: the first describes the socio-demographic characteristics of the sample, while the second presents the questionnaire responses related to participants’ perceptions and attitudes toward craft NABLAB.

### 3.1. Socio-Demographic Characteristics of the Sample

The total sample consisted of 527 participants who were recruited through a convenience sampling approach. The sample showed a balanced gender distribution (53.1% female and 46.9% male). The generational breakdown indicated a predominance of Millennials (35.3%), followed by Generation X (25.8%), Baby Boomers (19.5%), and Generation Z (19.4%). The educational level was generally high, with most respondents holding a master’s or specialist degree (33.6%), followed by those with a bachelor’s degree (20.9%) and a high school diploma (27.5%). A smaller proportion held a junior high school diploma (2.8%) or a postgraduate qualification (15.2%).

The final sample consisted of individuals who voluntarily chose to participate in the survey, reflecting a self-selected convenience population.

A detailed summary of the socio-demographic characteristics is reported in Table 2.

### 3.2. Questionnaire Results

Responses to the questionnaire allowed an analysis of participants’ knowledge, attitudes, and consumption habits regarding craft NABLAB. Overall, 48.2% of respondents indicated they would consider purchasing a craft NABLAB, while 51.8% would not. Purchase intention was higher among Baby Boomers (55.3%) compared to other generations.

About one-third of the sample (34.7%) reported having previously tried craft NABLAB, with higher rates among Millennials (36.6%) and Baby Boomers (39.8%), while Generation Z (29.4%) had the lowest consumption rate.

The perceived availability of these products was generally low: only 22.2% of respondents believed craft NABLAB is easy to find. The lowest perception of availability was among Generation X (18.4%), while Generation Z had the highest level of uncertainty, with nearly half of respondents (48.0%) saying they did not know.

When told that craft beers may contain beneficial compounds potentially good for health and supporting physical activity, 55.0% of respondents said they were more motivated to buy them. This motivation was highest among Baby Boomers (67.0%) and lowest among Millennials (50.0%).

Considering the harmful effects of alcohol, 54.1% of participants said they would prefer to buy a craft NABLAB rather than a traditional one, with Baby Boomers showing the greatest willingness (62.1%) and Generation X the least (50.7%).

Regarding price perception, 61.9% of respondents indicated they would be willing to pay a higher price for a craft NABLAB. This trend was more noticeable among Millennials (62.4%) and Baby Boomers (65.0%), while Generation X (55.1%) seemed less willing.

Only 13.9% of the total sample had consumed craft NABLAB at private social events, with slightly higher figures among Baby Boomers (18.4%). About a third of respondents (29.8%) believed that drinking these beers for their health- and activity-related benefits is becoming a trend, with higher percentages among Millennials (32.8%). Finally, 68.3% of participants said they would recommend craft NABLAB to friends or family interested in their potential functional properties, with the highest recommendation rates among Baby Boomers (74.8%) and Generation Z (74.5%).

A detailed breakdown of the responses by generational cohort is presented in Table 3.

To assess whether the responses to the questionnaire were independent of the participant’s generation, a Chi-square (χ^2^) Test of Independence was performed for each question. The results, including the Chi-square (χ^2^) statistic, degrees of freedom (*df*), *p*-value, and Cramér’s V Coefficient, are summarized in Table 4.

The inferential analysis revealed a statistically significant association (*p*-value ≤ 0.05) between generation and the responses to two specific questions: “Is it easy to find craft NABLAB beer?” and “Knowing that craft beer contains beneficial compounds (…) would you be more motivated to buy it?”. For all remaining questions, the null hypothesis of independence could not be rejected (*p*-value > 0.05).

Although these two items showed statistically significant associations, the Cramér’s V Coefficients calculated for all questions were consistently low (V < 0.13), indicating that the effect of generation on response patterns was weak.

## 4. Discussion

The current study offers new insights into Italian consumers’ perceptions of craft NABLAB, with a specific focus on generational differences in attitudes toward these emerging products. Overall, the findings show that although a growing number of consumers recognize the potential health-related and functional benefits of craft beer, market penetration and familiarity with craft NABLAB remain limited. Less than one-third of respondents reported having previously tried these products, and only one-fifth said they are easy to find in the market. Still, most people were positive about their potential advantages and said they would recommend them to others interested in wellness and nutrition.

These results align with previous research indicating that curiosity about innovative or health-focused beverages is growing, especially when such products are positioned within the broader context of functional foods and sustainability [70,71]. Similarly to trends seen in other European countries, Italian consumers seem driven by perceptions of authenticity, naturalness, and craftsmanship—traits closely linked to the craft beer movement [72]. However, the ongoing adherence to traditional drinking habits continues to restrict the adoption of craft NABLAB among regular beer drinkers, as also highlighted in studies on functional or low-alcohol wine and beer alternatives [73,74].

The intergenerational analysis reveals moderate but uneven interest in craft NABLAB. Although the sample overall is almost evenly split between those willing and not willing to purchase these products, differences across cohorts emerge. Baby Boomers appear the most inclined to consider purchasing (55.3%), whereas Gen Z, Millennials, and Gen X show more homogeneous and lower values. Similarly, previous consumption follows the same trend: Millennials and Baby Boomers report the highest prevalence of prior craft NABLAB consumption (36.6% and 39.8%, respectively), while Gen Z represents the group with the least direct experience (29.4%). This pattern suggests that interest in these beverages remains strongly linked to familiarity and prior experimentation.

Perceived accessibility of craft NABLAB is limited across all generations, with statistically significant though minimal differences as indicated by the Cramér’s V test (*p*-value < 0.05; Cramer’s V = 0.110). Only a minority of respondents believe the product is easy to find (22.2% overall), with slightly higher values among Millennials (24.7%) and lower among Gen X (18.4%). A substantial proportion of participants also report not knowing whether the product is easy to find, particularly among Gen Z and Baby Boomers. This limited distributional awareness represents a major barrier to the diffusion of craft NABLAB and mirrors similar challenges identified in Southern Europe, where restricted availability and low consumer awareness hinder access [75].

The potential health-related role of low- or no-alcohol craft beer represents an important driver across generations, with a statistically significant yet small association (Cramér’s *p*-value < 0.05; V = 0.126). In the total sample, 55% state that the presence of bioactive compounds (antioxidant, anti-inflammatory, neuroprotective, etc.) and the possible support for physical activity would increase their motivation to purchase, with higher values among Baby Boomers (67%). This sensitivity to functional benefits is consistent with the growing interest in beverages perceived as nutraceuticals, as reported in studies on the health-promoting properties of malt and hops [76,77]. This dynamic is particularly relevant in Italy, where wine culture remains dominant and non-alcoholic beers are often perceived as drinkable substitutes rather than truly functional beverages. Theoretically, these findings align with the Health Belief Model, in which perceived benefits are key predictors of the adoption of health-oriented behaviors. Older generations, in particular, seem to attribute greater value to the reduction in alcohol load and to the functional contribution of these beverages, making them priority targets for wellness-oriented communication strategies. Marketing could leverage these perceptions by emphasizing the nutraceutical profile of the product, its bioactive compound content, and its potential role in physical activity contexts—especially in a market where such properties are not yet clearly recognized by the average consumer.

Across the subsequent survey items, a consistent pattern emerges across generations. Approximately half of the sample stated that they would purchase a craft NABLAB due to the reduction or elimination of alcohol, while an even larger proportion would be willing to pay a higher price than for a traditional beer (61.9% overall). No marked generational differences appear, suggesting that sensitivity to lower alcohol content is a shared concern among consumers. Millennials show patterns similar to previous studies, demonstrating that they tend to associate craft and non-alcoholic beverages with lifestyle values, sustainability, and innovation [73,74,78]. The use of craft NABLAB in private social settings remains very limited across all cohorts, indicating that the product is not yet perceived as a convivial beverage. Likewise, the perception of craft NABLAB as an emerging trend is low (29.8%), with minimal variation across generations. These findings align with evidence from international mindful-drinking movements such as Dry January [79] and Sober October [80], which show that alcohol reduction is a widespread behavior but often confined to individual or temporary contexts rather than reflecting a broader cultural redefinition of non-alcoholic beverages. Overall, these elements indicate that the adoption of craft NABLAB is not yet firmly rooted either as a social practice or as a perceived trend, but could benefit from marketing strategies focused on healthy lifestyles and mindful drinking approaches [81].

Willingness to recommend craft NABLAB to individuals interested in health and physical activity is relatively high (68.3% overall), with higher values in Gen Z and Baby Boomers—the latter likely due to greater health awareness and concern about alcohol-related risks [76]. This suggests that, although craft NABLAB is not yet part of consolidated habits, they are perceived as compatible with a healthy lifestyle and therefore “recommendable.” From a theoretical perspective, this behavior relates to the subjective norms described in the Theory of Planned Behavior [82,83]: the belief that others may benefit from the product increases one’s own inclination to view it as a positive choice. This reinforces the idea that communication emphasizing the product’s benefits and functional attributes represents a strategic pathway to fostering attitude change and increasing intention to use.

From a public health perspective, replacing traditional beer with craft NABLAB may support harm-reduction policies, as suggested by international studies [75]. However, it remains essential not to overlook the sensory and cultural dimensions of consumption, particularly in Mediterranean contexts. Communication strategies that highlight craftsmanship, local ingredients, and moderate enjoyment could facilitate product acceptance [84]. Moreover, the literature shows that emotional states influence beverage choice: positive affective states are associated with wine and beer consumption, whereas negative ones tend to drive preference for spirits [85]. This underscores the opportunity to position craft NABLAB as beverages linked to conviviality and well-being, integrating them into positive and socially shared consumption experiences rather than presenting them as merely restrictive alternatives.

### Limitations and Future Research

This study provides valuable insights into the Italian consumer landscape for craft NABLAB; however, its findings should be considered in light of several limitations. The sample, although it exceeded the minimum size requirement, was recruited through a convenience sampling method, which may affect the generalizability of the results. This approach, combined with the online dissemination strategy, likely introduced a self-selection bias, potentially over-representing respondents with a pre-existing interest in craft beer, health, and wellness topics. Moreover, the recruitment channels used—such as social media, university networks, and craft beer communities—likely contributed to an over-representation of younger and highly educated individuals, further amplifying the self-selection bias and limiting the applicability of the findings to the broader Italian population. Additionally, the voluntary nature of participation further limits representativeness, as individuals less engaged with these topics may have been under-represented. Furthermore, the data are reliant on self-reported measures, which are susceptible to social desirability bias and the well-documented gap between stated intentions and actual purchasing behavior.

These limitations delineate clear and productive avenues for future research. A logical next step would be to replicate this study with a larger, probabilistically drawn sample. Extending this research to other countries with distinct beer cultures or similarly strong viticultural traditions—such as France, where the craft beer market is also experiencing significant growth—would allow for insightful cross-cultural comparisons and significantly enhance the external validity of the findings. The research design could be further strengthened by incorporating experimental or longitudinal approaches. For instance, future studies could assess how direct exposure—through controlled tasting sessions or detailed information on the nutraceutical properties of specific ingredients—influences actual purchase behavior and sensory perception, thereby moving beyond stated intentions.

As underscored by the present results, the emotional and contextual dimensions of consumption are crucial. Future research should probe deeper into these aspects, particularly as emotional states have been shown to mediate alcohol-related choices [85]. A particularly promising line of inquiry would be to investigate whether non-alcoholic craft beers can evoke the same positive emotional associations—such as conviviality and relaxation, as wine or traditional beer, or if they are perceived differently due to the lack of alcohol. Employing blind tastings would be a robust methodology to disentangle the influence of cognitive biases (e.g., the “alcohol-free” label) from genuine sensory perception, potentially revealing a greater appreciation for craft NABLAB than self-reports indicate.

Furthermore, while our study identified a general interest in the health and functional properties of craft NABLAB, we did not specifically target or segment consumers based on their level of physical activity or engagement in sports. Given the emerging evidence on the potential benefits of non-alcoholic beer for exercise recovery and rehydration [46], future research would greatly benefit from focusing on this specific consumer segment. For instance, studies could investigate the acceptance of craft NABLAB among amateur and professional athletes, or examine how messaging that directly links the product’s nutraceutical properties (e.g., polyphenols, electrolytes) to tangible exercise benefits (e.g., reduced muscle soreness, improved recovery) influences purchasing intent and sensory perception in a controlled, experimental setting.

Finally, building upon the growing interest in functional foods, future research should extend beyond consumer perception to empirically compare the nutraceutical profiles of traditional craft beers with their non-alcoholic counterparts. Moreover, exploring the application of novel probiotic yeasts or the addition of specific health-promoting ingredients (e.g., spices, herbs) could open new frontiers for developing craft NABLAB with enhanced functional properties. This aligns with evolving nutritional frameworks like the Modern Mediterranean Diet, which is increasingly inclusive of a wider range of fermented beverages [86,87].

## 5. Conclusions

This study explored Italian consumers’ perceptions of craft NABLAB across different generations, highlighting their potential positioning as functional beverages. The findings indicate growing interest and curiosity toward craft NABLAB, especially when these products are presented as healthier and more suitable alternatives for active lifestyles. However, limited market availability and persistent cultural associations between beer and alcohol still represent significant barriers to wider acceptance.

Baby Boomers and Millennials emerged as the most receptive groups—Baby Boomers due to greater health awareness, and Millennials for their openness to innovation and sustainability. Generation Z showed promising curiosity, though limited by lower familiarity with the product category.

From a public health and nutritional perspective, promoting the functional and nutraceutical properties of craft NABLAB could support more moderate and conscious drinking behaviors, encouraging a cultural shift toward reducing alcohol consumption while maintaining the social and sensory appeal of beer.

## Figures and Tables

**Table 1:** Advantages of craft NABLAB for Athletes.

Feature	Benefit for Athletes
No Alcohol	Avoids the diuretic effect, impaired sleep, and suppression of muscle protein synthesis associated with regular alcohol use [44]
Electrolytes	Support rehydration, muscle function, and prevention of cramps during recovery [45]
Polyphenols	Potential anti-inflammatory effects and immune system support [46]
Carbohydrates (Dextrins)	Helps with glycogen repletion after exercise [19]
Taste/Ritual	Provides the refreshing, satisfying taste of beer and social enjoyment without compromising performance or recovery [47]

**Table 2:** Personal details of study participants (*n* = 527).

Variable	Value, *n* (%)
Sex	
Female	280 (53.1)
Male	247 (46.9)
Generation	
Generation Z (born 1997–2012)	102 (19.4)
Millennials (1981–1996)	186 (35.3)
Generation X (1965–1980)	136 (25.8)
Baby Boomers (1946–1964)	103 (19.5)
Educational qualification	
Junior high school diploma	15 (2.8)
High school diploma	145 (27.5)
Bachelor’s degree	110 (20.9)
Master’s degree/specialist degree	177 (33.6)
Postgraduate degree	80 (15.2)

**Table 3:** Responses to the questionnaire on perceptions and attitudes toward craft non-alcoholic and low-alcohol beers (NABLAB) by generational cohort (*n* = 527). Data are presented as frequency (*n*) and percentage (%).

Questions	Generation Z (*n* = 102) *n* (%)	Millennials (*n* = 186) *n* (%)	Generation X (*n* = 136) *n* (%)	Baby Boomers (*n* = 103) *n* (%)	Total (*n* = 527) *n* (%)
Would you consider buying craft NABLAB beer?					
Yes	49 (48.0)	85 (45.7)	63 (46.3)	57 (55.3)	**254 (48.2)**
No	53 (52.0)	101 (54.3)	73 (53.7)	46 (44.7)	**273 (51.8)**
Have you ever consumed craft NABLAB beer?					
Yes	30 (29.4)	68 (36.6)	44 (32.4)	41 (39.8)	**183 (34.7)**
No	72 (70.6)	118 (63.4)	92 (67.6)	62 (60.2)	**344 (65.3)**
Is it easy to find craft NABLAB beer?					
Yes	23 (22.6)	46 (24.7)	25 (18.4)	23 (22.3)	**117 (22.2)**
No	30 (29.4)	76 (40.9)	45 (33.1)	27 (26.2)	**178 (33.8)**
I don’t know	49 (48.0)	64 (34.4)	66 (48.5)	53 (51.5)	**232 (44.0)**
Knowing that craft beer contains beneficial compounds (anti-inflammatory, neuroprotective, antioxidant, nutritional) and may support physical activity, would you be more motivated to buy it?					
Yes	57 (55.9)	93 (50.0)	71 (52.2)	69 (67.0)	**290 (55.0)**
No	45 (44.1)	93 (50.0)	65 (47.8)	34 (33.0)	**237 (45.0)**
Considering the harmful effects of alcohol, would you buy craft NABLAB beer?					
Yes	56 (54.9)	96 (51.6)	69 (50.7)	64 (62.1)	**285 (54.1)**
No	46 (45.1)	90 (48.4)	67 (49.3)	39 (37.9)	**242 (45.9)**
Would you pay more for a craft NABLAB beer than for a traditional one?					
Yes	68 (66.7)	116 (62.4)	75 (55.1)	67 (65.0)	**326 (61.9)**
No	34 (33.3)	70 (37.7)	61 (44.9)	36 (35.0)	**201 (38.1)**
Have you ever served craft NABLAB beer at private social events (e.g., dinners, parties)?					
Yes	12 (11.8)	26 (14.0)	16 (11.8)	19 (18.4)	**73 (13.9)**
No	90 (88.2)	160 (86.0)	120 (88.2)	84 (81.6)	**454 (86.1)**
Do you think the consumption of craft NABLAB beer for its health and physical activity properties is becoming a trend?					
Yes	31 (30.4)	61 (32.8)	37 (27.2)	28 (27.2)	**157 (29.8)**
No	71 (69.6)	125 (67.2)	99 (72.8)	75 (72.8)	**370 (70.2)**
Would you recommend craft NABLAB beer to friends or relatives interested in its health and physical activity properties?					
Yes	76 (74.5)	123 (66.1)	84 (61.8)	77 (74.8)	**360 (68.3)**
No	26 (25.5)	63 (33.9)	52 (38.2)	26 (25.2)	**167 (31.7)**

Footprint: Data relating to the total sample are shown in bold.

**Table 4:** Chi-square test results for survey responses on the perception, consumption, and purchase intentions of craft NABLAB (Non-Alcoholic and Low-Alcoholic Beer). For each item, the chi-square (χ^2^) statistic, degrees of freedom (*df*), *p*-value (with values ≤ 0.05 indicating statistical significance), and Cramér’s V Coefficient (measuring the strength of association) are reported.

Questions	χ^2^	*df*	*p*-Value	Cramér’s V Coefficient
Would you consider buying craft NABLAB beer?	2.76	3	0.430	0.072
Have you ever consumed craft NABLAB beer?	3.06	3	0.383	0.076
Is it easy to find craft NABLAB beer?	**12.74**	**6**	**0.047**	**0.110**
Knowing that craft beer contains beneficial compounds (anti-inflammatory, neuroprotective, antioxidant, nutritional) and may support physical activity, would you be more motivated to buy it?	**8.32**	**3**	**0.040**	**0.126**
Considering the harmful effects of alcohol, would you buy craft NABLAB beer?	3.79	3	0.285	0.085
Would you pay more for a craft NABLAB beer than for a traditional one?	4.06	3	0.255	0.088
Have you ever served craft NABLAB beer at private social events (e.g., dinners, parties)?	2.69	3	0.441	0.071
Do you think the consumption of craft NABLAB beer for its health and physical activity properties is becoming a trend?	1.59	3	0.662	0.055
Would you recommend craft NABLAB beer to friends or relatives interested in its health and physical activity properties?	6.89	3	0.076	0.114

Footprint: Statistically significant data are shown in bold.

## Data Availability

The data that support the findings of this study are available from the corresponding author, M.R., upon reasonable request. The data are not publicly available due to privacy and ethical restrictions.
